# Cryptic diversity and range extension in the big-eyed bat genus *Chiroderma* (Chiroptera, Phyllostomidae)

**DOI:** 10.3897/zookeys.918.48786

**Published:** 2020-03-12

**Authors:** Burton K. Lim, Livia O. Loureiro, Guilherme S. T. Garbino

**Affiliations:** 1 Department of Natural History, Royal Ontario Museum, 100 Queen’s Park, Toronto, Ontario M5S 2C6, Canada Royal Ontario Museum Toronto Canada; 2 Department of Ecology and Evolutionary Biology, University of Toronto, Toronto, Ontario M5S 3B2, Canada University of Toronto Toronto Canada; 3 PPG-Zoologia, Departamento de Zoologia, Instituto de Ciências Biológicas, Universidade Federal de Minas Gerais, Avenida Antônio Carlos 6627, Pampulha, 31270-901, Belo Horizonte, Minas Gerais, Brazil Universidade Federal de Minas Gerais Belo Horizonte Brazil

**Keywords:** *Chiroderma gorgasi*, *Chiroderma improvisum*, cryptic species, cytochrome c oxidase subunit 1, Lesser Antilles

## Abstract

Since the last systematic review of *Chiroderma* (big-eyed bats) more than two decades ago, we report on biodiversity surveys that expand the distribution and species diversity of this Neotropical genus. The Caribbean endemic species *Chiroderma improvisum* is documented for the first time from Nevis in the northern Lesser Antilles. A broader geographic sampling for a molecular analysis identifies a paraphyletic relationship in *Chiroderma trinitatum* with respect to *Chiroderma doriae*. Cis-Andean populations of *C. trinitatum* are most closely related to the morphologically distinctive and allopatrically distributed *C. doriae* in the Cerrado and Atlantic Forest of Brazil and Paraguay. The sister taxon to this grouping includes trans-Andean populations of *C. trinitatum*, which we recommend to elevate to species status as *C. gorgasi*. This is an example of a cryptic species because *C. gorgasi* was previously considered morphologically similar to *C. trinitatum*, but more detailed examination revealed that it lacks a posterolabial accessory cusp on the lower second premolar and has a narrower breadth of the braincase. We provide an amended description of *Chiroderma gorgasi*.

## Introduction

Cryptic species, phenotypically similar organisms that are classified as a single species but are genetically divergent lineages, are being discovered at a greater rate due to the increasing prevalence of molecular methods, such as DNA barcoding (e.g., Hebert et al. 2004). It has been estimated that Neotropical mammalian biodiversity is underestimated by one-third (Lim 2012). At typical lowland tropical forest sites, bats comprise the majority of mammal species diversity (Voss and Emmons 1996), so more species are expected to be recognized in this group as traditional taxonomic hypotheses are tested by genetic techniques. In addition, new surveying methods such as the use of triple-high netting systems to catch higher flying aerial insectivorous bats, and harp traps to target species that may be able to better detect mist nets, is decreasing the sampling bias associated with traditional mist nets set just above ground level.

The big-eyed bats in the genus *Chiroderma* Peters (Phyllostomidae) are characterized by greatly reduced nasal bones in the skull and a combination of external features including a white dorsal stripe that does not extend onto the head; legs and interfemoral membrane conspicuously hairy; and relatively large eyes (Straney 1984; Gardner 2008). The genus currently comprises six species (Simmons 2005, Taddei and Lim 2010): *C. doriae* Thomas, 1891 occurs in central-eastern Brazil and Paraguay; *C. improvisum* Baker & Genoways, 1976 is endemic to the Lesser Antillean islands of Guadeloupe, Montserrat, and Saint Kitts (Beck et al. 2016); *C. salvini* Dobson, 1878 is found from Mexico to Bolivia (recent records from Brazil are misidentifications of *C. villosum* Peters, 1860 – see Brandão et al. 2019); *C. trinitatum* Goodwin, 1958 is distributed from Honduras (Turcios-Casco et al. 2020) and Costa Rica to Amazonian Brazil and Trinidad; *C. villosum* ranges from Mexico to southeastern Brazil and Trinidad; and *C. vizottoi* Taddei & Lim, 2010 is found only in northeastern Brazil.

The systematics of *Chiroderma* was last reviewed by Baker et al. (1994) based on a phylogenetic study of the mitochondrial DNA cytochrome *b* (Cytb) gene; however, each of the five species known at the time was represented by a single specimen. With broader geographic coverage, we re-assess the distributional range, genetic diversity, and morphological differences in the genus.

## Material and methods

### Fieldwork

We conducted a survey of bats on the Caribbean island of Nevis from 24–29 April 2016. Live traps used included a harp trap and 6 m or 12 m mist nets set singly in the forest understory or on a triple-high telescoping pole system. Traps were regularly monitored for the first 2–3 hours after sunset when bat activity is the highest after they leave their roosts to feed. Individuals not kept as part of the representative collection documenting the species diversity were released at point of capture. A combined scientific research and export permit (F002) was issued through the authority of the Nevis Historical and Conservation Society. An Animal Use Protocol (2016-01) was obtained from the Royal Ontario Museum Animal Care Committee. An import permit (#2016-02101-4) was authorized by the Canadian Food Inspection Agency. Use of wild mammals followed the guidelines of the American Society of Mammalogists (Sikes et al. 2016).

### Molecular analyses

The cytochrome c oxidase subunit 1 (CO1) gene is the best represented molecular marker for *Chiroderma* on the genetic sequence database GenBank (www.ncbi.nih.gov/genbank). There are 117 samples from nine countries in Central and South America (Brazil, Ecuador, El Salvador, French Guiana, Guatemala, Guyana, Mexico, Panama, and Suriname). We add 26 new sequences to bring the sample size to 143 sequences representing 12 countries in the Neotropics, including Venezuela, Peru, and Nevis, and five species in the genus (Appendix 1). There are no tissue samples or nucleotide sequences on GenBank of any genes for the recently described *Chiroderma vizottoi* (Taddei and Lim 2010). Outgroup taxa were other genera in the subtribe Vampyressina Baker et al., 2016 (*Platyrrhinus incarum* Thomas, 1912 and *Uroderma bilobatum* Peters, 1866) of the New World leaf-nosed bats, for direct comparison to Baker et al. (1994) in their analysis of Cytb. Alternative phylogenetic relationships within the subtribe are given by Baker et al. (2016) and Rojas et al. (2016). We also analyzed Cytb, but there are only 11 sequences on GenBank, although we did add one new sequence of *Chiroderma trinitatum gorgasi* from Panama (Appendix 2).

Molecular methods for new sequences of CO1 follow the protocol for DNA extraction, PCR amplification, and automated nucleotide sequencing outlined in Lim (2017). For Cytb, extraction, amplification, and sequencing followed Lim et al. (2008). Base calls were confirmed with bidirectional sequences and aligned using Sequencher version 4.8 (Gene Code Corporation, Ann Arbor, Michigan). Phylogenetic and molecular evolutionary analyses were conducted using MEGA version 6 (Tamura et al. 2013). For a robust comparison of phylogeny, we used parsimony as a method that minimizes evolutionary change without an explicit model of evolution and maximum likelihood as a probabilistic method with an explicit model of evolution. Maximum parsimony used the subtree pruning regrafting inference method with 500 bootstrap replicates to test branch supports. Maximum likelihood used the Tamura 3-parameter substitution model and gamma distributed rates with invariant sites for COI as determined by the best fit test. For Cytb, the Tamura Nei model and gamma rates were the best fit. Tree inference used nearest neighbor interchange heuristic inference with 500 bootstrap replicates. Genetic distances were calculated with the Tamura 3-parameter model with gamma distributed rates among sites for the larger COI dataset.

### Morphological analyses

Morphological and morphometric comparisons included 138 specimens from five species of *Chiroderma*, including two *C. improvisum*, four *C. doriae*, seven *C. salvini*, 58 *C. trinitatum*, and 66 *C. villosum* (Appendix 3). We also analyzed the holotypes of *C. trinitatum gorgasi* Handley, 1960 and *C. trinitatum trinitatum* Goodwin, 1958, but did not have specimens of the most recently described *C. vizottoi*. Only adults (defined as having closed cranial sutures and complete epiphyseal ossification of metacarpal and phalanx joints) of both sexes were examined. Specimens are deposited in the following institutions; Royal Ontario Museum (ROM, Toronto, Canada); National Museum of Natural History (USNM, Washington, DC, USA); American Museum of Natural History (New York, USA); Texas Tech University (Lubbock, USA); and Field Museum of Natural History (Chicago, USA).

Measurements defined below were taken with digital calipers accurate to 0.01 mm following the descriptions of Handley (1960): forearm length (FA); greatest length of skull (GSL); interorbital width (IOW); postorbital width (POW); braincase width (BCW); condyloincisive length (CIL); zygomatic breadth (ZB); width across upper molars (M-M); width across upper canines (C-C); and length of maxillary toothrow (C-M). An analysis of variance (ANOVA) for each measurement and a multivariate analysis of variance (MANOVA) were performed to examine the significance of morphometric divergence among species of *Chiroderma*. The level of significance was *p* = 0.05 for all statistical tests. The homoscedasticity of each variable was tested using Bartlett’s test with the R package mvoutliers. Statistical analyzes were performed using R 3.1.0 (R Core Team 2005) and PAST 2.17. Variables were log-transformed and a correlation matrix was used in a Principal Components Analysis (PCA) to assess phenetic differences in multivariate morphological space.

## Results

**Figure 1.**
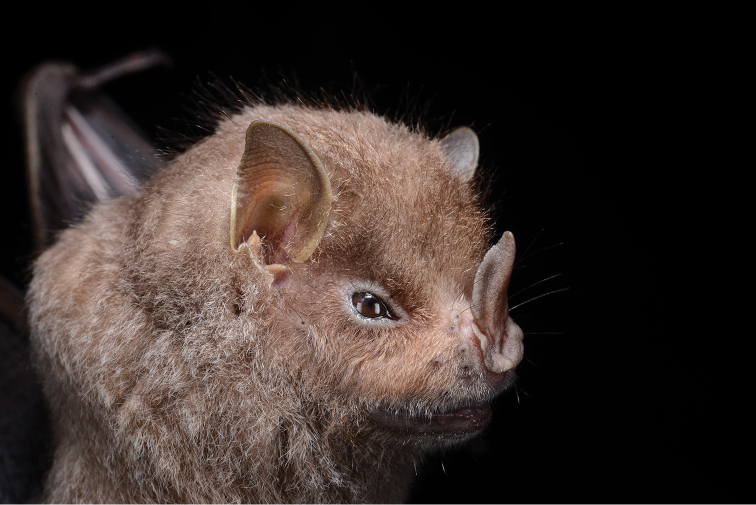
First record of the big-eyed bat *Chiroderma improvisum* from Nevis (ROM 126002).

**Figure 2.**
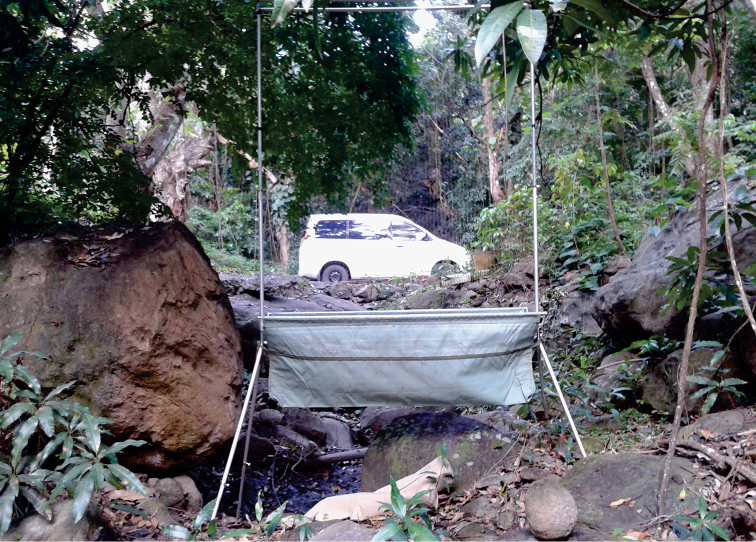
Collecting locality of the first record of the big-eyed bat *Chiroderma improvisum* from Nevis caught in a harp trap on April 28, 2016. The habitat is a dry ravine within forest that is bisected by a road in the residential area of Barnes Ghaut.

We report the first occurrence of *Chiroderma improvisum* (Fig. 1) from Nevis in the northern Leeward Islands of the Lesser Antilles in the Caribbean. An adult male was caught at Barnes Ghaut on April 28, 2016, in a harp trap set across a dry ravine in forest bisected by a road and surrounded by residential homes (Fig. 2). Other equipment deployed included 6 m mist nets set on a triple-high telescoping pole system, a single 6 m mist net, and a single 12 m mist net from 1900–2100 h. In addition to the new distributional record for the island, one *Ardops nichollsi*, one *Noctilio leporinus*, and 12 *Artibeus jamaicensis* were captured.

### Molecular analyses

**Figure 3.**
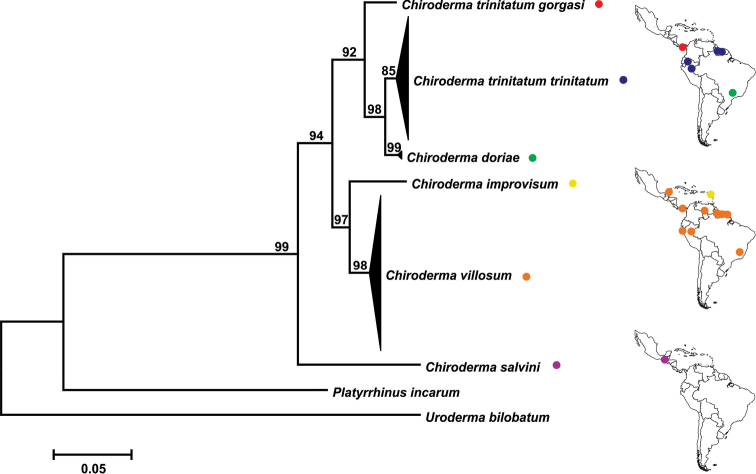
Maximum likelihood tree of cytochrome c oxidase subunit 1 gene for big-eyed bats *Chiroderma* and general localities of molecular sampling sites. Bootstrap percentages show support at each node.

For COI, the 657 basepairs (bp) at the 5’ end were available for most (82%) of the specimens analyzed. The complete 1140 bp of Cytb were available, including the newly generated sequence, for most (75%) of the specimens analyzed. The topology of the *Chiroderma* COI maximum likelihood tree identified six primary terminal clades with (1) *C. salvini* as sister species to all other taxa; (2) *C. improvisum* and (3) *C. villosum* as sister species; and (4) *C. doriae* sister to (5) *C. trinitatum trinitatum* with (6) *C. trinitatum gorgasi* sister to these taxa (Fig. 3; Suppl. material 1: Fig. S1). These phylogenetic relationships were supported by bootstrap values ≥85 and were congruent with the maximum parsimony tree (Suppl. material 2: Fig. S2), which had bootstrap values ≥73. Not surprisingly for linked mtDNA loci, the same interspecific topology was recovered by the smaller Cytb dataset analyzed by maximum likelihood (Suppl. material 3: Fig. S3) and maximum parsimony (Suppl. material 4: Fig. S4), except for lower bootstrap supports. The unexpected result was the paraphyly of *C. trinitatum* in relation to *C. doriae*. The sister-group relationship of *C. t. trinitatum* and *C. doriae* was well supported by values ≥73 in all molecular analyses.

Interspecific genetic distances of the larger COI dataset ranged from 11.3% between *C. doriae* and *C. salvini* to 2.5% between *C. doriae* and *C. t. trinitatum* (Table 1). The sequence divergence between *C. t. trinitatum* and *C. t. gorgasi* was 3.9%. Intraspecific distances were 1% within *C. villosum*, 0.9% within *C. t. trinitatum*, and 0.2% within *C. doriae*, but three taxa were represented by only one sample.

**Table 1.** Genetic divergence of cytochrome c oxidase subunit 1 for the big-eyed bat *Chiroderma* and outgroup taxa *Uroderma* and *Platyrrhinus*. Interspecific distances shown in the lower left matrix; intraspecific distances shown in bold in the diagonal.

	(1)	(2)	(3)	(4)	(5)	(6)	(7)	(8)
*U. bilobatum* (1)	–							
*P. incarum* (2)	0.203	–						
*C. villosum* (3)	0.223	0.178	**0.010**					
*C. improvisum* (4)	0.231	0.194	0.047	–				
*C. t. trinitatum* (5)	0.222	0.184	0.067	0.075	**0.009**			
*C. salvini* (6)	0.205	0.185	0.093	0.101	0.110	–		
*C. t. gorgasi* (7)	0.195	0.149	0.059	0.070	0.039	0.101	–	
*C. doriae* (8)	0.213	0.173	0.066	0.077	0.025	0.113	0.039	**0.002**

### Morphological analyses

**Figure 4.**
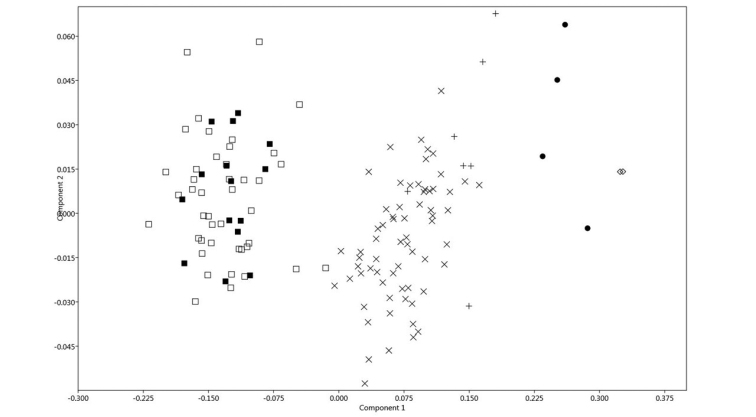
Principal Component Analysis (PCA) carried out using the correlation matrix of ten measurements for six taxa of the big-eyed bat *Chiroderma*. *C. trinitatum gorgasi* (■) *C. trinitatum trinitatum* (□), *C. salvini* (×), *C. villosum* (+), *C. doriae* (●), and *C. improvisum* (◊).

Cranial and body measurements for the six taxa of *Chiroderma* identified in the molecular analyses are shown in Table 2. *Chiroderma trinitatum gorgasi* and *C. trinitatum trinitatum* are the smallest members of the genus, whereas *C. improvisum* is the largest for most measurements. In the PCA, there are three main groups of species (Fig. 4). The first group is formed by the smaller taxa *C. t. gorgasi* and *C. t. trinitatum*. The second group has species with medium size, *C. villosum* and *C. salvini*, and the third group is formed by the largest species of the genus, *C. doriae* and *C. improvisum*. The first and second principal components (PC1 and PC2) explained 94.5% of the total variation. PC1 shows a pattern in general size variation and is explained mostly by C-M, C-C, and FA. PC2 has positive loadings for most measurements, especially IOW, with the exception of C-M, C-C, M-M, and ZB that have negative loadings (Table 3). All the species seem to occupy the entire range of PC2, indicating that the contrast among measurements is negligible and it is not responsible for the separation of groups.

**Table 2.** Cranial and body measurements of six taxa of the big-eyed bat *Chiroderma*. See Material and methods for variable abbreviations.

	*C. trinitatum gorgasi* (*N* = 11)	*C. trinitatum trinitatum* (*N* = 47)	*C. villosum* (*N* = 66)	*C. salvini* (*N* = 6)	*C. doriae* (*N* = 4)	*C. improvisum* (*N* = 2)
FA	37.7 (37.0–40.5)	38.9 (37.1–42.6)	47.9 (44.6–51.0)	49.6 (49.3–50.0)	54 (53.0–55.0)	58.2 (56.2–60)
GLS	21.2 (20.6–21.7)	21.1 (20.0–22.7)	24.5 (23.2–25.7)	26.1 (24.2–26.5)	28.2 (27.6–28.9)	29.3 (28.7–29.9)
CLI	17.3 (16.3–18.1)	17.4 (16.1–18.8)	20.3 (18.7–21.58)	21.6 (21.5–22.3)	23.9 (23.1–24.5)	26.3 (25.3–27.8)
ZB	13.0 (12.5–13.5)	12.9 (11.7–14.2)	15.5 (14.4–16.7)	16.2 (15.8–16.7)	17.84 (17.7–18.1)	18.75 (18.5–19)
POW	5.3 (4.9–5.6)	5.3 (5.8–5.8)	5.9 (5.3–6.3)	6.2 (5.9–6.3)	6.4 (6.1–6.6)	6.6 (6.5–6.6)
IOW	5.6 (5.2–5.9)	5.5 (5.0–6.2)	6.0 (5.5–6.8)	6.8 (6.1–7.3)	7.6 (7.1–7.8)	7.4 (7.4–7.4)
BCW	9.4 (8.9–9.8)	9.6 (9.2–10.4)	10.7 (10.1–11.5)	11.21 (11.0–11.5)	11.9 (11.3–12.1)	12.0 (11.5–12.5)
C-M	7.0 (6.5–7.3)	7.1 (6.7–7.8)	8.7 (8.1–9.4)	9.4 (9.1–9.4)	10.3 (10.0–11.1)	11.0 (10.9–11.1)
M-M	9.6 (9.2–10.0)	9.5 (8.7–10.3)	11.3 (10.3–12.4)	12.0 (11.5–12.3)	13.3 (13.0–13.7)	13.8 (13.6–13.9)
C-C	4.7 (4.4–5.0)	4.6 (4.1–5.0)	5.8 (5.3–6.3)	6.1 (6.0–6.2)	6.5 (6.3–6.8)	7.4 (7.4–7.4)

**Table 3.** Eigenvalue and loadings for the first and second components in the Principal Component Analysis (PCA) of big-eyed bats *Chiroderma*. See Material and methods for variable abbreviations.

	PC1	PC2
Eigenvalue	1.57	0.53
% Variance	91.4	3.11
IOW	0.25	0.44
POW	0.21	0.40
C-M	0.34	-0.23
GSL	0.31	-0.05
BCW	0.34	0.35
CIL	0.34	0.07
C-C	0.36	-0.23
M-M	0.36	-0.14
ZB	0.37	-0.12
FA	0.42	0.32

All variables had *p* > 0.05 for Bartlett’s test of homoscedasticity, indicating constant variances (*p* values: FA = 0.06, GSL = 0.25, IOW = 0.59, POW = 0.3l, BCW = 0.06, CIL = 0.45, ZB = 0.08, M-M = 0.32, C-C = 0.08, and C-M = 0.06). The MANOVA and the ANOVA demonstrated that *C. t. trinitatum* and *C. t. gorgasi* are significantly different from the other taxa of *Chiroderma* (P < 0.001) for all measured variables (Appendix 3). By contrast, *C. t. gorgasi* and *C. t. trinitatum* are not significantly different from each other (*p* = 0.16, *F* = 56.0). However, the ANOVA showed that one measurement, BCW (*p* = 0.01; *F* = 62.0), was significantly larger for *C. t. trinitatum* than for *C. t. gorgasi*. All other cranial measurements had smaller mean values for *C. t. trinitatum* than for *C. t. gorgasi*.

**Figure 5.**
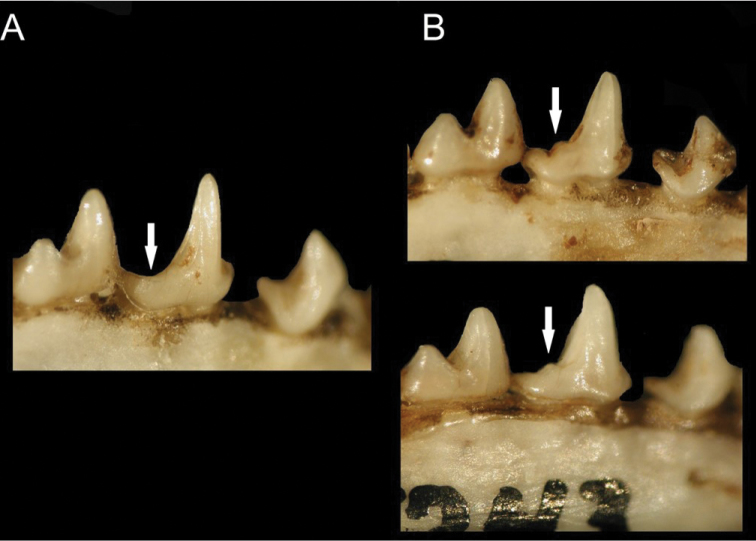
Lateral view of the second lower premolar on the right mandible of **A***Chiroderma gorgasi* and **B***C. trinitatum*. The arrow points to the accessory cusp that is absent in *C. gorgasi* and present in *C. trinitatum*. But note the variation in cusp formation in *C. trinitatum*.

Although similar in size, *C. t. trinitatum* has a more robust breadth of the braincase than *C. t. gorgasi*. *Chiroderma t. trinitatum* also has an accessory cusp on the second lower premolar, which is absent in *C. t. gorgasi* (Fig. 5). In the genetic analyses, *C. t. trinitatum* is well supported as the sister species to *C. doriae* and does not share a most recent common ancestor with *C. t. gorgasi*. We consider this as a previous example of a cryptic species and therefore now recognize *C. gorgasi* as a distinct species from *C. trinitatum*. Sáez and Lozano (2005: 111) considered cryptic species to be “groups of organisms that are morphologically indistinguishable from each other, yet found to belong to different evolutionary lineages”. They also stated that “after detailed comparisons of morphological and non-morphological features, we can often establish key morphological characters for their identification. In those cases, we can then refer to pseudo-cryptic or pseudo-sibling species”. Because Handley’s original description was qualitative and univariate, we offer an amended description of this taxon.

### Taxonomic account

#### Chiroderma gorgasi

Taxon classificationAnimaliaChiropteraPhyllostomidae

Handley, 1960

FFD367BC-610F-510F-B5C9-4C7F370CFDE3

Chiroderma gorgasi Handley, 1960:464
Chiroderma trinitatum gorgasi Barriga-Bonilla, 1965:246

##### Material examined.

***Holotype.*** – USNM 309903 (Field number COH 5436), adult male with skin, skull and partial skeleton. Collected on March 6, 1959, by C. O. Handley, Jr, and B. R. Feinstein in Tacarcuna Village (8°05'N, 77°17'W), 3200 feet [975 meters], Río Pucro, Darién, Panama.

##### Geographic distribution.

**Figure 6.**
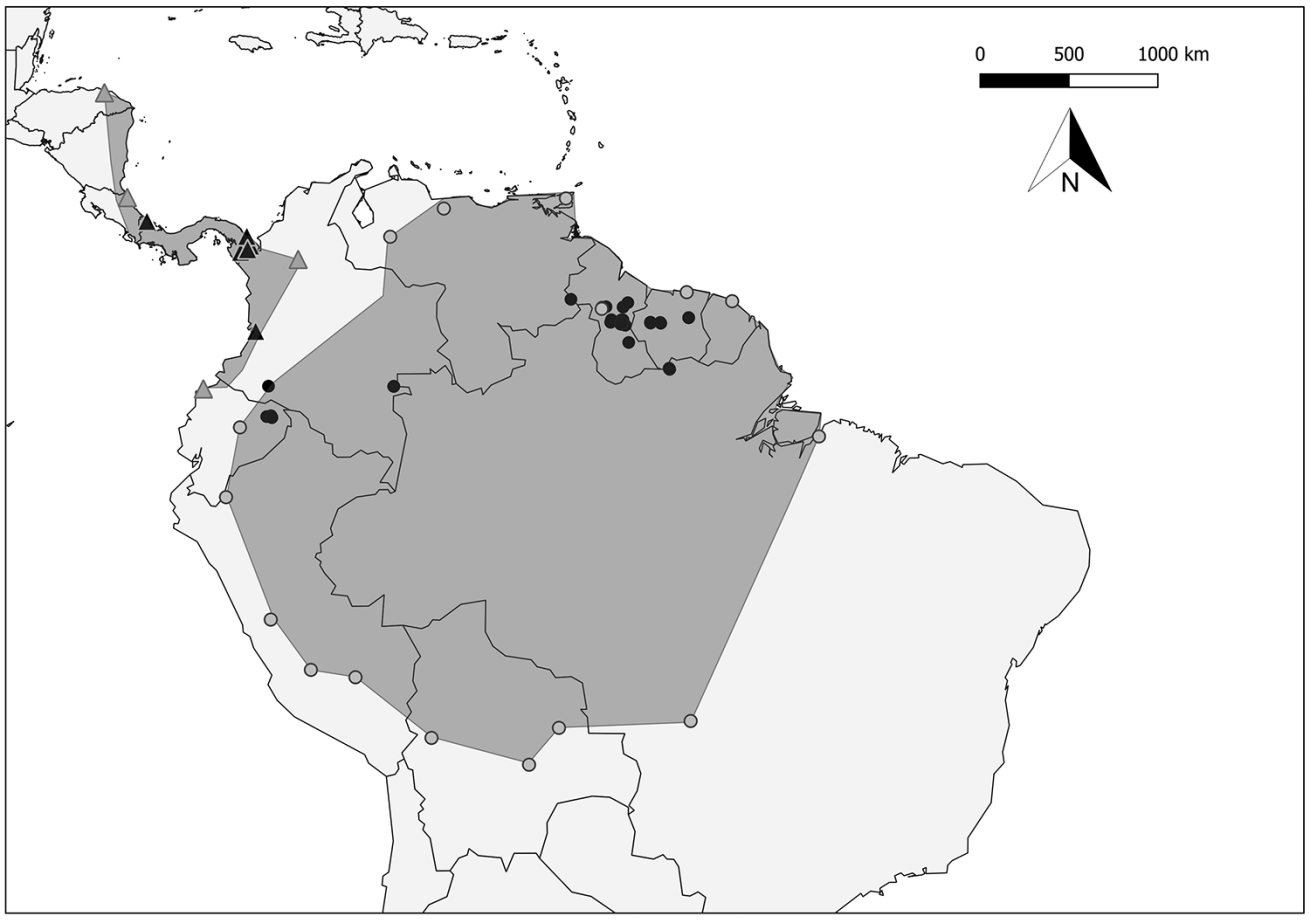
Geographic distribution of *Chiroderma gorgasi* (▲) and *C. trinitatum* (●) localities analyzed in our study (See Appendix 3) (gray circle) Represents marginal localities reported for *C. trinitatum* and (gray triangle) *C. gorgasi* reported by previous papers (Handley 1967; Pine et al. 1970; Ojasti and Linares 1971; Gardner 1976; Albuja 1989; Timm and LaVal 1998; Lim and Engstrom 2001; Genoways et al. 1981; Webster and Fugler 1984; Anderson 1997; Ochoa et al. 1988; Simmons and Voss 1998; Gardner 2008).

*Chiroderma gorgasi* is distributed west of the Andes in northwestern Ecuador (Albuja 1989), western Colombia (Gardner 2008), Panama (Handley 1960), Costa Rica (LaVal and Rodríguez-Herrera 2002), and Honduras (Turcios-Casco et al. 2020) (Fig. 6).

##### Description.

**Figure 7.**
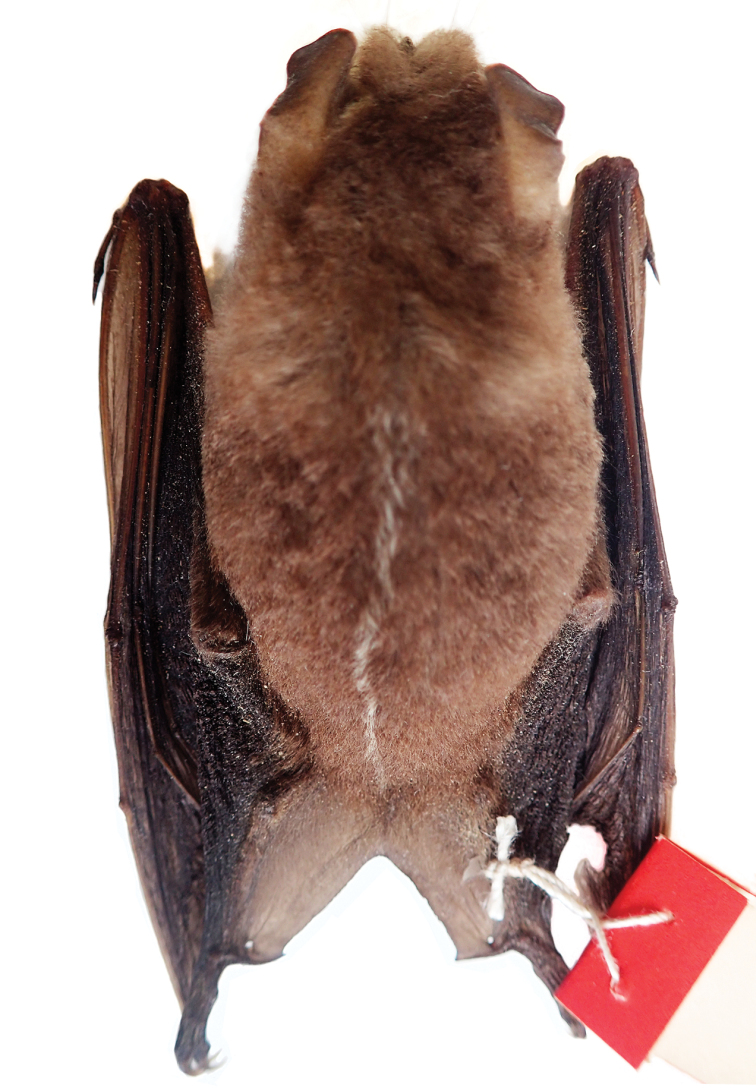
Dorsal view of the skin of the holotype of *Chiroderma gorgasi* (USNM 309903).

**Figure 8.**
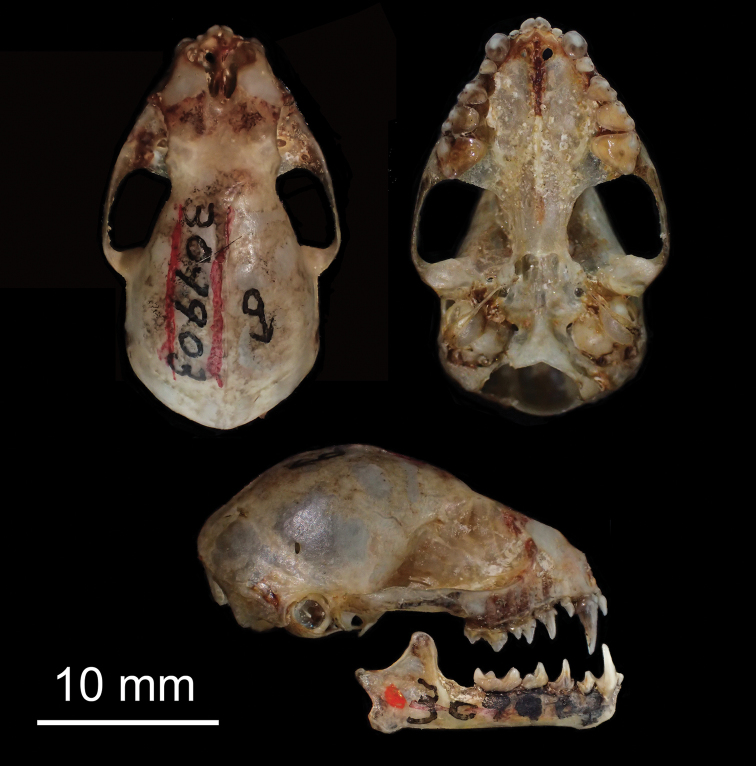
Dorsal, ventral, and lateral views of the skull of the holotype of *Chiroderma gorgasi* (USNM 309903).

*Chiroderma gorgasi* is a small species of *Chiroderma* (FA 37.0–40.5; GLS 20.2–22.5) that is similar in size to *C. trinitatum* (sensu stricto) (Table 2). Overall, the dorsal pelage is tricolor varying from light to dark brown (Fig. 7). The dorsal hairs have a dark brown band at the base, a buff coloration in the middle, and brown tips. A white medial stripe extends from the interscapular region to the base of the rump. Proximal two-thirds of forearm hairy. Basal third of uropatagium hairy. Conspicuous white facial stripes extend from the noseleaf to the inner base of the ears, and from the posterior part of the upper lip to the base of the ears. The uropatagium is medium brown. The skull has an elongated braincase with an undeveloped sagittal and lambdoidal crest. The nasal aperture is short, not extending beyond the second premolar. The occipital is rounded in posterior view. The upper incisors are thin and elongated with parallel or convergent tips, which may or may not touch apically. The second lower premolar lacks a third cusp (Fig. 5). The postorbital processes are undeveloped and rounded (Fig. 8).

##### Comparisons.

*Chiroderma gorgasi* is morphologically very similar to *C. trinitatum*. Both species have a small cranial and body size for the genus (Table 2, Fig. 4), an undeveloped sagittal and lambdoidal crest, a rounded occipital complex, a short nasal aperture, and undeveloped supraorbital region. However, *C. trinitatum* has a third posterior cusp on the second lower premolar, which is absent in *C. gorgasi* (Fig. 5). This cusp in *C. trinitatum* may vary from very pointed and developed to rounded and less marked, but is always present. In addition, *C. gorgasi* tends to have a broader braincase (Table 4) and a flatter supraorbital region, which tends to be deeper in *C. trinitatum*.

**Table 4.** ANOVA comparing *Chiroderma trinitatum trinitatum* to *C. doriae*, *C. improvisum*, *C. villosum*, *C. salvini*, and *C. trinitatum gorgasi*. * indicates significantly distinct variables (*p* ≤ 5%).

Variables	*C. doriae*	*C. improvisum*	*C. villosum*	*C. salvini*	*C. t. gorgasi*
GSL	< 2.2e-16*	< 2.2e-16*	< 2.2e-16*	< 2.2e-16*	0.1541
CI	< 2.2e-16*	< 2.2e-16*	< 2.2e-16*	< 2.2e-16*	0.4423
ZB	< 2.2e-16*	< 2.2e-16*	< 2.2e-16*	< 2.2e-16*	0.3094
POW	< 2.2e-16*	1.373e-06*	< 2.2e-16*	1.061e-12*	0.6747
IOW	< 2.2e-16*	1.347e-09*	2.366e-14*	1.355e-14*	0.6272
BWC	< 2.2e-16*	2.602e-08*	< 2.2e-16*	< 2.2e-16*	0.0339*
M-C	< 2.2e-16*	< 2.2e-16*	< 2.2e-16*	< 2.2e-16*	0.5257
B-M	< 2.2e-16*	1.527e-15*	< 2.2e-16*	< 2.2e-16*	0.1444
B-C	< 2.2e-16*	< 2.2e-16*	< 2.2e-16*	< 2.2e-16*	0.1251
FA	< 2.2e-16*	< 2.2e-16*	< 2.2e-16*	< 2.2e-16*	0.1753

*Chiroderma gorgasi* is easily distinguished from other species of the genus by its smaller cranial and body size (Table 2). *C. villosum* shares with *C. gorgasi* an elongated braincase, rounded occipital region in dorsal view, and absence of a third cusp on the second lower premolar. However, *C. gorgasi* has an undeveloped postorbital processes, a short nasal aperture, and conspicuous white stripes on the face and back, whereas *C. villosum* has a very developed and pointed postorbital processes, a long nasal aperture, which extends beyond the first molar, a conspicuous posterior palatine spine, and usually incipient white stripes on the face and back. *Chiroderma salvini* resembles *C. gorgasi* in the undeveloped sagittal and lambdoidal crest and by the rounded postorbital processes, but a set of other cranial characters distinguish both species, such as a triangular occipital complex and a long nasal aperture. In the dentition, *C. gorgasi* can be readily distinguished from *C. salvini* and *C. villosum* by having a tall first lower premolar, with a crown height approximately 2/3 the height of the crown of the second lower premolar, and placed approximately in the middle of the distance between the canine and the second lower premolar. In *C. salvini* and *C. villosum*, this tooth is much smaller, usually with a low crown, shorter than the mesiodistal length of the tooth, and is nearer to the canine than to the second lower premolar.

*Chiroderma doriae* and *C. improvisum* are the largest species of the genus, and unlike *C. gorgasi* have a triangular occipital complex in dorsal view, a pointed and developed supraorbital region, a relatively more developed sagittal and lambdoidal crest, and a long nasal aperture. In addition, *C. doriae* also tends to have a relatively broader braincase than *C. gorgasi* and the presence of an undeveloped third cusp in the second lower premolar. We were not able to examine specimens of the more recently described *C. vizottoi*, but it is larger than *C. gorgasi* and most similar to *C. doriae* in qualitative craniodental traits.

## Discussion

The only big-eyed bat species occurring in the Caribbean is *Chiroderma improvisum*, which until recently was known from Guadeloupe (Baker and Genoways 1976) and Montserrat (Jones and Baker 1979; Pierson et al. 1986) by six individuals (Larsen et al. 2007). Subsequently, it was caught on Saint Kitts by Beck et al. (2016) and we are the first to report its occurrence on Nevis. Although this species has been sporadically documented since its discovery, the distribution has broadened in the northern Lesser Antilles but this may be ephemeral depending on weather systems such as hurricanes (Larsen et al. 2007).

*Chiroderma gorgasi* was originally described by Handley (1960) using five specimens from the type locality in Panama. The author distinguished the new species from *C. trinitatum* by its smaller size, deeper brain case, shorter rostrum, shaper lacrimal ridge, bulging forehead, larger upper incisors, and thicker white band in the dorsal hairs. But at that time, *C. trinitatum* was only known by the holotype from Trinidad (Goodwin 1958) so the extent of variation within each species was poorly understood. Based on a specimen from Mitu in Amazonian Colombia, Barriga-Bonilla (1965) recognized the taxon as two subspecies and assigned his Colombian specimen to *C. t. gorgasi*. The subspecies were considered to be distributed from eastern Panama to western Venezuela for *C. t. gorgasi* and Trinidad to the Amazon basin for *C. t. trinitatum* (Jones and Carter 1976). However, with more geographic sampling the initial distinctions between the two taxa were less obvious due to individual and geographic variation (Simmons and Voss 1998), as also demonstrated by our morphometric analysis. But the taxonomy and distributional limits were still contentious with Gardner (2008) recognizing the Andes as the delineation of the subspecies and reassigning the specimen of Barriga-Bonilla (1965) from Mitu, Colombia, to *C. t. trinitatum*.

Our morphological review identified the presence of three cusps on the second lower premolar in cis-Andean populations referable to *C. trinitatum* and two cusps in trans-Andean populations referable to *C. gorgasi* that also match the taxonomic boundaries of Gardner (2008). Morphometrically, *C. trinitatum* averages smaller than *C. gorgasi* in all cranial measurements except for a proportionately broader braincase. Furthermore, our genetic analyses recovered *C. trinitatum* as the well-supported sister species to the larger and morphologically distinctive *C. doriae*, and not to the superficially similar *C. gorgasi*. Based on this morphological and molecular evidence, we recognize *C. gorgasi* as a distinct species and divergent lineage that does not share the most recent common ancestor with *C. trinitatum* (sensu stricto).

The overall topology of the Cytb tree proposed by Baker et al. (1994) is identical to our tree except for the recognition of *C. gorgasi*, which they did not have a sample of, as the sister species to *C. trinitatum* and *C. doriae*. The evolution of *Chiroderma* was suggested as occurring primarily by allopatric speciation (Baker et al. 1994). More specifically, *C. improvisum* arose by peripatric speciation in the Lesser Antilles after dispersing from its most recent common ancestor with *C. villosum* in South America. The Andes is an obvious geographic barrier separating *C. gorgasi* from the most recent common ancestor of *C. trinitatum* and *C. doriae*. A dated phylogeny is needed to test whether this is an older sundering event associated with the uplift of the northern Andes in the Late Miocene or a more recent dispersal event followed by isolation and the cessation of gene flow. Rojas et al. (2016) date the divergence of *Chiroderma* species to the Pliocene-Holocene, but *C. gorgasi* was not included in their dataset. The allopatric distribution of *C. trinitatum* and *C. doriae* suggests that perhaps the Cerrado Savanna in Brazil acted as a barrier after colonization of the Atlantic Forest from the Amazon, but the records of *C. doriae* for the Cerrado and the discovery of a species of *Chiroderma* in the dry deciduous forests of the Brazilian Caatinga, *C. vizottoi*, indicates that species of the genus can adapt to more harsh habitats. The speciation event that gave rise to *C. salvini* and the most recent common ancestor of the other species of *Chiroderma* is speculative without a thorough biogeographic analysis with a dated phylogeny.

Although not an overly species-rich genus, biodiversity surveys and molecular analyses are finding new distributional and taxonomic discoveries in *Chiroderma*. However, there are still large geographic gaps in sampling throughout the Neotropics, such as the Amazon basin in Brazil and northern South America in Colombia and Venezuela. In addition, this has hindered detailed study of the biogeography of the genus and more broadly the evolution of bats in the Neotropics.

## Supplementary Material

XML Treatment for Chiroderma gorgasi

## Appendix 1

**Table:** Tissue samples of *Chiroderma* used in the cytochrome c oxidase subunit 1 analysis.

Sample ID	Species	GenBank	Country	State/Department
ROM 111114	*Chiroderma doriae*	JF448016	Brazil	Sao Paulo
ROM 111141	*Chiroderma doriae*	JF446371	Brazil	Sao Paulo
ROM 111149	*Chiroderma doriae*	JF446373	Brazil	Sao Paulo
ROM 111163	*Chiroderma doriae*	JF446372	Brazil	Sao Paulo
	*Chiroderma villosum*	KT236232	Brazil	Espirito Santo
	*Chiroderma villosum*	KT236233	Brazil	Espirito Santo
ROM 105191	*Chiroderma trinitatum*	JF448017	Ecuador	Napo
ROM 105230	*Chiroderma trinitatum*	JF448810	Ecuador	Napo
ROM 105243	*Chiroderma trinitatum*	JF448806	Ecuador	Napo
ROM 105253	*Chiroderma trinitatum*	JF448805	Ecuador	Napo
ROM 105581	*Chiroderma trinitatum*	JF448811	Ecuador	Napo
ROM 105685	*Chiroderma trinitatum*	JF448807	Ecuador	Napo
ROM 105718	*Chiroderma trinitatum*	JF448809	Ecuador	Napo
ROM 105766	*Chiroderma trinitatum*	JF448808	Ecuador	Napo
ROM 106342	*Chiroderma trinitatum*	JF448812	Ecuador	Napo
ROM F40504	*Chiroderma trinitatum*	JF448813	Ecuador	Napo
ROM 104448	*Chiroderma villosum*	JF448818	Ecuador	Napo
ROM 104540	*Chiroderma villosum*	JF448829	Ecuador	Napo
ROM 104541	*Chiroderma villosum*	JF448814	Ecuador	Napo
ROM 104549	*Chiroderma villosum*	JF448828	Ecuador	Napo
ROM 105244	*Chiroderma villosum*	JF448815	Ecuador	Napo
ROM 105254	*Chiroderma villosum*	JF448816	Ecuador	Napo
ROM 105267	*Chiroderma villosum*	JF448826	Ecuador	Napo
ROM 105361	*Chiroderma villosum*	JF448825	Ecuador	Napo
ROM 105540	*Chiroderma villosum*	JF448824	Ecuador	Napo
ROM 105587	*Chiroderma villosum*	JF448830	Ecuador	Napo
ROM 105719	*Chiroderma villosum*	JF448822	Ecuador	Napo
ROM 105720	*Chiroderma villosum*	JF448821	Ecuador	Napo
ROM 105721	*Chiroderma villosum*	JF448820	Ecuador	Napo
ROM 105928	*Chiroderma villosum*	JF448817	Ecuador	Napo
ROM 105968	*Chiroderma villosum*	JF448819	Ecuador	Napo
ROM F37400	*Chiroderma villosum*	JF448827	Ecuador	Napo
ROM F37774	*Chiroderma villosum*	JF448823	Ecuador	Napo
ROM 101245	*Chiroderma villosum*	JF446499	El Salvador	Ahuachapan
	*Chiroderma villosum*	KU295490	French Guiana	
ROM 99703	*Chiroderma salvini*	JF446777	Guatemala	El Progreso
ROM 103486	*Chiroderma trinitatum*	JF454560	Guyana	Upper Demerara-Berbice
ROM 103503	*Chiroderma trinitatum*	JF454561	Guyana	Upper Demerara-Berbice
ROM 103504	*Chiroderma trinitatum*	MN714876	Guyana	Upper Demerara-Berbice
ROM 103505	*Chiroderma trinitatum*	JF454562	Guyana	Upper Demerara-Berbice
ROM 107205	*Chiroderma trinitatum*	EF080285	Guyana	Potaro-Siparuni
ROM 107419	*Chiroderma trinitatum*	EF080286	Guyana	Potaro-Siparuni
ROM 107476	*Chiroderma trinitatum*	MN714877	Guyana	Potaro-Siparuni
ROM 108144	*Chiroderma trinitatum*	JF454552	Guyana	Cuyuni-Mazaruni
ROM 108244	*Chiroderma trinitatum*	JF454559	Guyana	Cuyuni-Mazaruni
ROM 108463	*Chiroderma trinitatum*	JF454544	Guyana	Potaro-Siparuni
ROM 108554	*Chiroderma trinitatum*	JF454545	Guyana	Potaro-Siparuni
ROM 108587	*Chiroderma trinitatum*	JF454555	Guyana	Potaro-Siparuni
ROM 108588	*Chiroderma trinitatum*	JF454554	Guyana	Potaro-Siparuni
ROM 108714	*Chiroderma trinitatum*	EF080287	Guyana	Potaro-Siparuni
ROM 108763	*Chiroderma trinitatum*	MN714878	Guyana	Potaro-Siparuni
ROM 108889	*Chiroderma trinitatum*	EF080288	Guyana	Potaro-Siparuni
ROM 108950	*Chiroderma trinitatum*	JF454557	Guyana	Potaro-Siparuni
ROM 108993	*Chiroderma trinitatum*	JF454556	Guyana	Potaro-Siparuni
ROM 109026	*Chiroderma trinitatum*	MN714879	Guyana	Potaro-Siparuni
ROM 109195	*Chiroderma trinitatum*	JF454558	Guyana	Potaro-Siparuni
ROM 109271	*Chiroderma trinitatum*	JF454553	Guyana	Potaro-Siparuni
ROM 109333	*Chiroderma trinitatum*	JF454542	Guyana	Potaro-Siparuni
ROM 111627	*Chiroderma trinitatum*	JF454543	Guyana	Potaro-Siparuni
ROM 111809	*Chiroderma trinitatum*	JF454547	Guyana	Potaro-Siparuni
ROM 111844	*Chiroderma trinitatum*	MN714880	Guyana	Potaro-Siparuni
ROM 111884	*Chiroderma trinitatum*	JF454546	Guyana	Potaro-Siparuni
ROM 111946	*Chiroderma trinitatum*	JF454548	Guyana	Potaro-Siparuni
ROM 115807	*Chiroderma trinitatum*	JF454550	Guyana	Potaro-Siparuni
ROM 116630	*Chiroderma trinitatum*	JF454549	Guyana	Potaro-Siparuni
ROM 118996	*Chiroderma trinitatum*	JF454551	Guyana	Upper Takutu-Upper Essequibo
ROM 121975	*Chiroderma trinitatum*	MN714881	Guyana	Potario-Siparuni
ROM 125124	*Chiroderma trinitatum*	MN714882	Guyana	Potaro-Siparuni
ROM 103214	*Chiroderma villosum*	JF454584	Guyana	Upper Takutu-Upper Essequibo
ROM 103331	*Chiroderma villosum*	JF454585	Guyana	Upper Takutu-Upper Essequibo
ROM 106644	*Chiroderma villosum*	JF454566	Guyana	Upper Takutu-Upper Essequibo
ROM 107111	*Chiroderma villosum*	EF080290	Guyana	Potaro-Siparuni
ROM 107112	*Chiroderma villosum*	EF080291	Guyana	Potaro-Siparuni
ROM 107394	*Chiroderma villosum*	EF080292	Guyana	Potaro-Siparuni
ROM 108203	*Chiroderma villosum*	JF454565	Guyana	Cuyuni-Mazaruni
ROM 108219	*Chiroderma villosum*	JF454564	Guyana	Cuyuni-Mazaruni
ROM 108764	*Chiroderma villosum*	JF454571	Guyana	Potaro-Siparuni
ROM 108765	*Chiroderma villosum*	JF454570	Guyana	Potaro-Siparuni
ROM 108843	*Chiroderma villosum*	EF080289	Guyana	Potaro-Siparuni
ROM 108998	*Chiroderma villosum*	JF454573	Guyana	Potaro-Siparuni
ROM 109138	*Chiroderma villosum*	JF454572	Guyana	Potaro-Siparuni
ROM 109175	*Chiroderma villosum*	JF454569	Guyana	Potaro-Siparuni
ROM 109221	*Chiroderma villosum*	JF454568	Guyana	Potaro-Siparuni
ROM 109270	*Chiroderma villosum*	JF454567	Guyana	Potaro-Siparuni
ROM 109307	*Chiroderma villosum*	JF454583	Guyana	Potaro-Siparuni
ROM 109308	*Chiroderma villosum*	JF454582	Guyana	Potaro-Siparuni
ROM 109337	*Chiroderma villosum*	JF454581	Guyana	Potaro-Siparuni
ROM 111628	*Chiroderma villosum*	EF080293	Guyana	Potaro-Siparuni
ROM 111629	*Chiroderma villosum*	JF459119	Guyana	Potaro-Siparuni
ROM 111754	*Chiroderma villosum*	JF454580	Guyana	Potaro-Siparuni
ROM 111768	*Chiroderma villosum*	JF454579	Guyana	Potaro-Siparuni
ROM 111769	*Chiroderma villosum*	JF454578	Guyana	Potaro-Siparuni
ROM 111770	*Chiroderma villosum*	JF454577	Guyana	Potaro-Siparuni
ROM 111788	*Chiroderma villosum*	JF454576	Guyana	Potaro-Siparuni
ROM 111836	*Chiroderma villosum*	JF454575	Guyana	Potaro-Siparuni
ROM 111845	*Chiroderma villosum*	JF454574	Guyana	Potaro-Siparuni
ROM 119167	*Chiroderma villosum*	MN714883	Guyana	Upper Takutu-Upper Essequibo
ROM 119230	*Chiroderma villosum*	JF454586	Guyana	Upper Takutu-Upper Essequibo
ROM 122481	*Chiroderma villosum*	MN714884	Guyana	Potaro-Siparuni
ROM 98850	*Chiroderma villosum*	JF454563	Guyana	Barima-Waini
ROM 125179	*Chiroderma villosum*	MN714885	Guyana	East Berbice-Corentyne
ROM F38952	*Chiroderma villosum*	MN714886	Guyana	Potaro-Siparuni
ROM 98702	*Uroderma bilobatum*	JF435925	Guyana	Barima-Waini
ROM 96536	*Chiroderma villosum*	JF448018	Mexico	Campeche
ROM FN30654	*Chiroderma villosum*	JF447242	Mexico	Campeche
ROM 104342	*Chiroderma gorgasi*	MN714901	Panama	Darien
ROM 104352	*Chiroderma villosum*	JF447405	Panama	Darien
ROM F38210	*Chiroderma villosum*	JF447406	Panama	Darien
ROM 122084	*Chiroderma trinitatum*	MN714887	Peru	Loreto
ROM 122137	*Chiroderma trinitatum*	MN714888	Peru	Loreto
ROM 122149	*Chiroderma trinitatum*	MN714889	Peru	Loreto
ROM 122165	*Chiroderma villosum*	MN714890	Peru	Loreto
ROM 122260	*Chiroderma villosum*	MN714891	Peru	Loreto
ROM 125567	*Chiroderma villosum*	MN714892	Peru	Tumbes
ROM 126002	*Chiroderma improvisum*	MN714893	Nevis	Saint Thomas Lowland Parish
ROM 114170	*Chiroderma trinitatum*	JF447622	Suriname	Brokopondo
ROM 114213	*Chiroderma trinitatum*	JF447625	Suriname	Brokopondo
ROM 114233	*Chiroderma trinitatum*	JF447623	Suriname	Brokopondo
ROM 114234	*Chiroderma trinitatum*	JF447624	Suriname	Brokopondo
ROM 117003	*Chiroderma trinitatum*	JF447627	Suriname	Sipaliwini
ROM 117003	*Chiroderma trinitatum*	MN714894	Suriname	Sipaliwini
ROM 117027	*Chiroderma trinitatum*	JF447626	Suriname	Sipaliwini
ROM 117083	*Chiroderma trinitatum*	JF447628	Suriname	Sipaliwini
ROM 117376	*Chiroderma trinitatum*	EU096695	Suriname	Sipaliwini
ROM 117555	*Chiroderma trinitatum*	EU096696	Suriname	Sipaliwini
ROM 120098	*Chiroderma trinitatum*	MN714895	Suriname	Sipaliwini
ROM 120168	*Chiroderma trinitatum*	HQ545629	Suriname	Sipaliwini
ROM 120225	*Chiroderma trinitatum*	HQ545678	Suriname	Sipaliwini
ROM 120384	*Chiroderma trinitatum*	HQ919736	Suriname	Sipaliwini
ROM 114212	*Chiroderma villosum*	JF447630	Suriname	Brokopondo
ROM 114228	*Chiroderma villosum*	JF447631	Suriname	Brokopondo
ROM 117119	*Chiroderma villosum*	JF447629	Suriname	Sipaliwini
ROM 117375	*Chiroderma villosum*	EU096697	Suriname	Sipaliwini
ROM 120226	*Chiroderma villosum*	HQ545679	Suriname	Sipaliwini
ROM 120239	*Chiroderma villosum*	HQ545445	Suriname	Sipaliwini
ROM 120240	*Chiroderma villosum*	HQ545446	Suriname	Sipaliwini
ROM 120354	*Chiroderma villosum*	MN714896	Suriname	Sipaliwini
ROM 120364	*Chiroderma villosum*	HQ919717	Suriname	Sipaliwini
ROM 121027	*Chiroderma villosum*	MN714897	Suriname	Sipaliwini
ROM 121117	*Chiroderma villosum*	MN714898	Suriname	Sipaliwini
ROM 126174	*Chiroderma villosum*	MN714899	Suriname	Para
ROM 113919	*Platyrrhinus incarum*	JF435616	Suriname	Brokopondo
ACUNHC 393	*Chiroderma villosum*	MN714900	Venezuela	Amazonas

## Appendix 2

**Table:** Tissue samples of *Chiroderma* used in the cytochrome b analysis.

Sample ID	Species	GenBank	Country	State/Province
UNESP 16506	*Chiroderma doriae*	L28937	Brazil	Sao Paulo
TK 16379	*Chiroderma doriae*	AY169958	Brazil	
TK 15713	*Chiroderma improvisum*	L28938	Montserrat	St. Anthony
TK 25052	*Chiroderma villosum*	DQ312414	Trinidad	St. George
FMNH 174652	*Chiroderma villosum*	FJ154121	Peru	Madre de Dios
TK 17627	*Platyrrhinus helleri*	L28940	Suriname	Marowijne
TK 25256	*Uroderma bilobatum*	L28941	Trinidad	St. George
TK 22581	*Chiroderma salvini*	L28939	Panama	Darien
TK 25211	*Chiroderma trinitatum*	DQ312413	Trinidad	St. George
ASK 7799	*Chiroderma villosum*	JF442196	Ecuador	Orellana
ASK 7667	*Chiroderma villosum*	JF442139	Ecuador	Napo
MN 36375	*Chiroderma villosum*	DQ903823	Brazil	
SK-Bat-61	*Chiroderma improvisum*	JQ915203	Saint Kitts	
ROM 104342	*Chiroderma gorgasi*	MN714902	Panama	Darien

## Appendix 3

Specimens of *Chiroderma* examined morphologically. Vouchers examined are arranged alphabetically by species and country. See “Material and Methods” for collection acronyms.

*Chiroderma doriae* – Brazil: São Paulo - ROM 111163, ROM 111141, ROM 111114, ROM 111149.

*Chiroderma improvisum* – Montserrat: St. Anthony Parsish – TTU 31403; St. Kitts and Nevis: Barnes Ghaut - ROM 126002.

*Chiroderma salvini* – El Salvador: Morazan - ROM 83365, ROM 85948, Santa Ana - ROM 101526; Guatemala: El Progreso - ROM 99703; Panama: Darien - ROM 78472, ROM 91194.

*Chiroderma trinitatum gorgasi* – Colombia: Valle del Cauca - USNM 483763, USNM 483765, Antioquia - USNM 499478, USNM 499476; Panama: Bocas Del Toro – USNM 319498, USNM 335295, Darien - FMNH 128132, ROM 104342, USNM 309901, USNM 309903-**holotype**, San Blas - USNM 309905.

*Chiroderma trinitatum trinitatum* – Colombia: Vaupes - ROM 45276, ROM 45278, ROM 45280, ROM 45281, ROM 45284, Putumayo – ROM 63236, ROM 63237, ROM 63238; Ecuador: Napo - ROM 105191, ROM 105243, ROM 105253, ROM 105685, ROM 105766, ROM 106342; Guyana: Cuyuni-Mazaruni - ROM 108144, Demerara-Berbice - ROM 57392, ROM 103486, ROM 103503, Upper, Potaro-Siparuni - ROM 107205, ROM 107419, ROM 107476, ROM 108463, ROM 108554, ROM 108587, ROM 108714, ROM 108763, ROM ROM 108889, ROM 108950, ROM 108993, ROM 109195, ROM 109333, ROM 111627, ROM 111809, ROM 111884, ROM 111946, ROM 115807, ROM 116630; Suriname: Brokopondo - ROM 114170, ROM 114213, ROM 114233, ROM 114234, Sipaliwini - ROM 117027, ROM 117376, ROM 120168, ROM 120225, ROM 120384; Trinidad: Saint Andrew County – AMNH 175325-**holotype**.

*Chiroderma villosum* – Bolivia: Carrasco - ROM 78471; Colombia: Choco - ROM 85849, Vaupes - ROM 44952, ROM 44953, ROM 44954, ROM 45243, ROM 45245, ROM 45246, ROM 45247, ROM 45249, ROM 45250, ROM 45251, ROM 45252, ROM 45253, ROM 45254, ROM 45255, ROM 45257; Ecuador: Napo - ROM 104448, ROM 104541, ROM 104549, ROM 105244, ROM 105254, ROM 105361, ROM 105720, ROM 105721); Guyana: Barima-Waini - ROM 98850, Potaro-Siparuni - ROM 107111, ROM 107112, ROM 107394, ROM 108219, ROM 108764, ROM 108843, ROM 108998, ROM 109138, ROM 109175, ROM 109221, ROM 109307, ROM 109308, ROM 109337, ROM 111628, ROM 111629, ROM 111754, ROM 111768, ROM 111769, ROM 111770, ROM 111788, ROM 111836, ROM 111845, ROM 122481, Upper Demerara-Berbice - ROM 60402, ROM 60423, Upper Takutu-Upper Essequibo - ROM 35614, ROM 103214, ROM 106644, ROM 119167, ROM 119230; Panama: Darien - ROM 104352; Suriname: Brokopondo - ROM 114212, Sipaliwini - ROM 117119, ROM 117375, ROM 120226, ROM 120239, ROM 120364, ROM 121027; Trinidad and Tobago: Nariva - ROM 124684, ROM 124691.

## Supplementary materials

Supplementary material 1 Maximum likelihood tree of cytochrome c oxidase subunit 1 gene for big-eyed bats *Chiroderma* as presented in Fig. 3, but expanded to show individuals Data type: phylogenetic dendrogram https://binary.pensoft.net/file/388746This dataset is made available under the Open Database License (http://opendatacommons.org/licenses/odbl/1.0/). The Open Database License (ODbL) is a license agreement intended to allow users to freely share, modify, and use this Dataset while maintaining this same freedom for others, provided that the original source and author(s) are credited.Burton K. Lim, Livia O. Loureiro, Guilherme S. T. Garbino

Supplementary material 2 Maximum parsimony tree of cytochrome c oxidase subunit 1 gene for *Chiroderma*. Bootstrap percentages show support at each node Data type: phylogenetic dendrogram https://binary.pensoft.net/file/388747This dataset is made available under the Open Database License (http://opendatacommons.org/licenses/odbl/1.0/). The Open Database License (ODbL) is a license agreement intended to allow users to freely share, modify, and use this Dataset while maintaining this same freedom for others, provided that the original source and author(s) are credited.Burton K. Lim, Livia O. Loureiro, Guilherme S. T. Garbino

Supplementary material 3 Maximum likelihood tree of cytochrome b gene for *Chiroderma*. Bootstrap percentages show support at each node Data type: phylogenetic dendrogram https://binary.pensoft.net/file/388748This dataset is made available under the Open Database License (http://opendatacommons.org/licenses/odbl/1.0/). The Open Database License (ODbL) is a license agreement intended to allow users to freely share, modify, and use this Dataset while maintaining this same freedom for others, provided that the original source and author(s) are credited.Burton K. Lim, Livia O. Loureiro, Guilherme S. T. Garbino

Supplementary material 4 Maximum parsimony tree of cytochrome b gene for *Chiroderma*. Bootstrap percentages show support at each node Data type: phylogenetic dendrogram https://binary.pensoft.net/file/388749This dataset is made available under the Open Database License (http://opendatacommons.org/licenses/odbl/1.0/). The Open Database License (ODbL) is a license agreement intended to allow users to freely share, modify, and use this Dataset while maintaining this same freedom for others, provided that the original source and author(s) are credited.Burton K. Lim, Livia O. Loureiro, Guilherme S. T. Garbino
