# Supplementary figures and images for: Cryptic diversity and range extension in the big-eyed bat genus Chiroderma (Chiroptera, Phyllostomidae)
Source: Zookeys. 2020 Mar 12;918:41–63. doi: 10.3897/zookeys.918.48786 (PMC7086341; doi:10.3897/zookeys.918.48786)

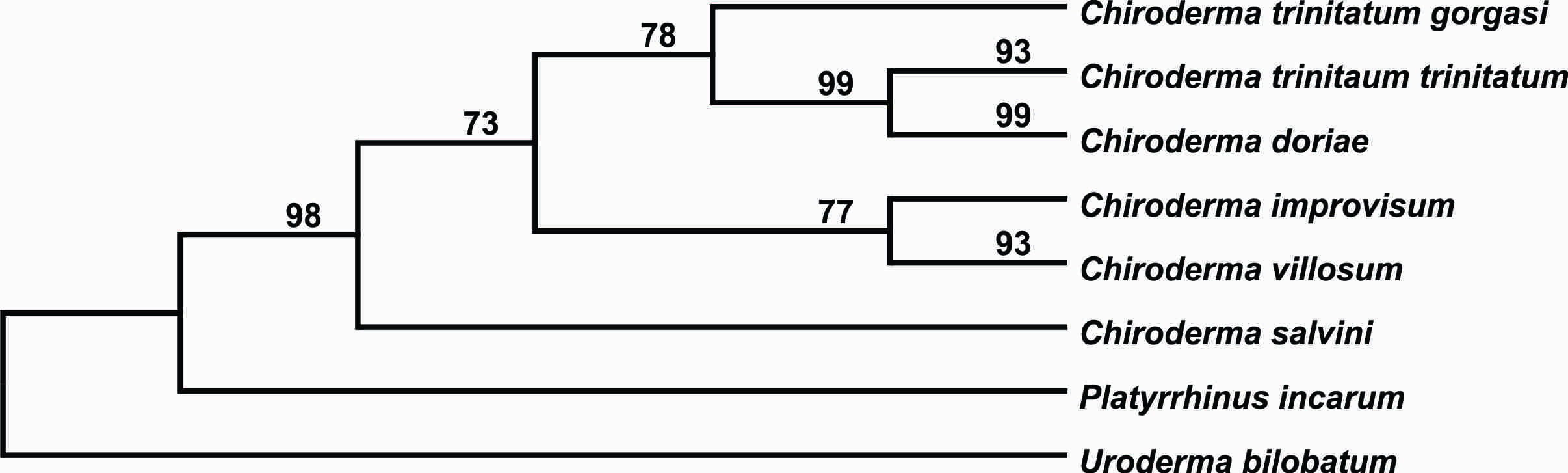

Supplement: Supplementary material 2 [file zookeys-918-041-s002.jpg]

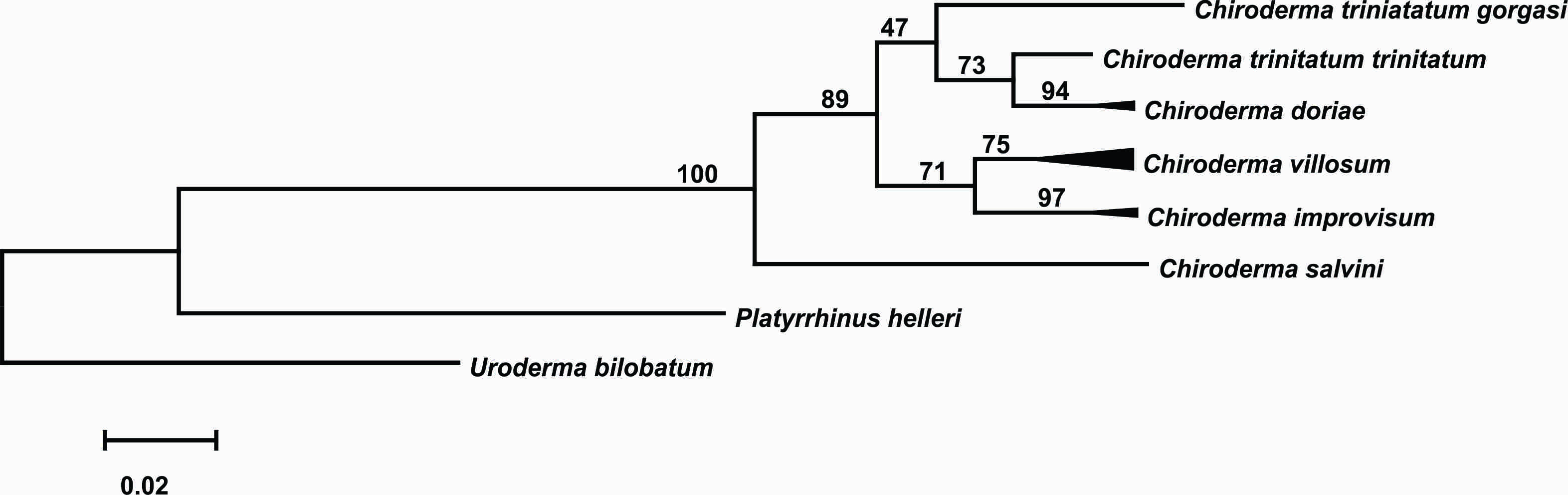

Supplement: Supplementary material 3 [file zookeys-918-041-s003.jpg]

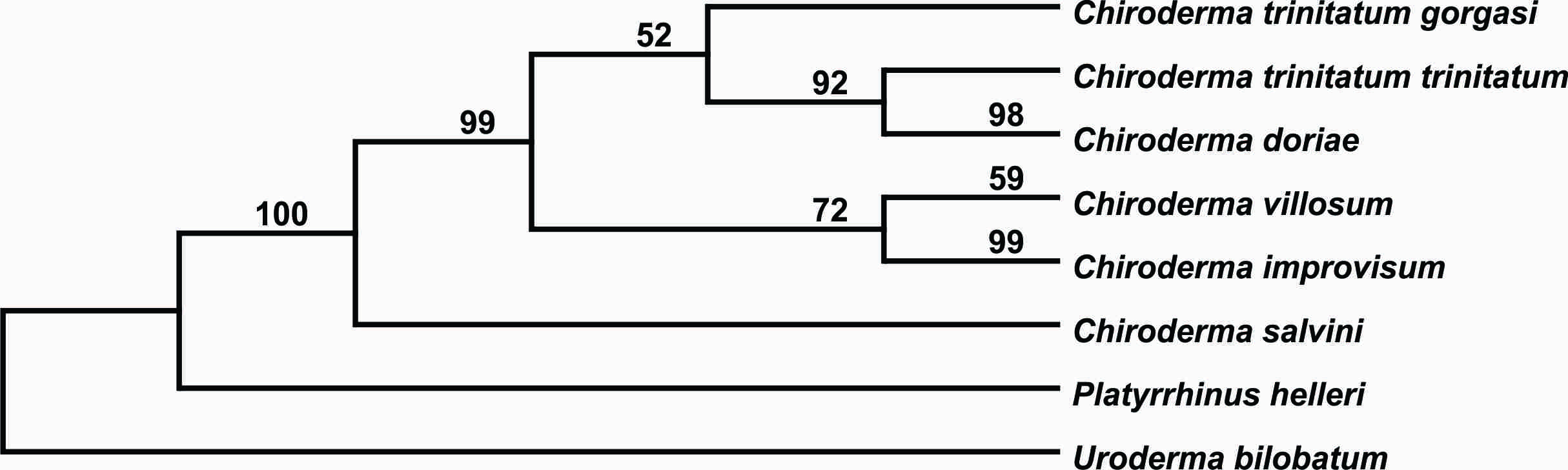

Supplement: Supplementary material 4 [file zookeys-918-041-s004.jpg]
